# Isocentric Navigation for the Training of Percutaneous Endoscopic Transforaminal Discectomy: A Feasibility Study

**DOI:** 10.1155/2018/6740942

**Published:** 2018-07-15

**Authors:** Guoxin Fan, Chaobo Feng, Wangcheng Xie, Dongdong Wang, Fei Liu, Chun Yuan, Zhi Zhou, Shisheng He

**Affiliations:** ^1^Orthopedics Department, Shanghai Tenth People's Hospital, Tongji University School of Medicine, Shanghai, China; ^2^Orthopedics Department, Zhangjiagang City Hospital of Traditional Chinese Medicine, Jiangsu, China

## Abstract

**Background:**

Percutaneous endoscopic transforaminal discectomy (PETD) is usually chosen for lumbar disc herniation due to its obvious advantages such as small incision and absence of nerve or muscular traction. However, learning PETD is a great challenge for inexperienced surgeons.

**Objective:**

The study aimed to investigate whether isocentric navigation would be beneficial in PETD training.

**Methods:**

A total of 117 inexperienced surgeons were trained with PETD at L2/3, L3/4, L4/5, and L5/S1 on the cadavers without (Group A n=58) or with (Group B n=59) isocentric navigation. Puncture times, fluoroscopy times, exposure time, and radiation dose were recorded and analyzed. Questionnaires were conducted before and after the training program.

**Result:**

Isocentric navigation could improve young surgeons' satisfaction with the training program and decrease the puncture times, fluoroscopy times, exposure time, and radiation dose significantly (P<0.001).

**Conclusion:**

Isocentric navigation contributes to the training of PETD and may improve its standardization, homogenization, and generalization.

## 1. Introduction

Symptomatic lumbar disc herniation (LDH) is considered as the chief culprit of low back pain and sciatic pain [1, 2]. Minimally invasive spine surgery (MISS) has been well validated for the surgical management of LDH [3, 4], which has been rapidly spread all around the world [5]. As a MISS technique, percutaneous endoscopic transforaminal discectomy (PETD) has noninferior efficacy to open microdiscectomy [6, 7] with merits of normal paraspinal structures preservation, minimal postoperative pain, low risk of postoperative epidural scar formation and iatrogenic instability, rapid recovery, short operation time, and low postoperative expenses [7–12].

However, learning PELD still remains as a great challenge for inexperienced surgeons [12–15], mainly because they need to build the spatial sense of planned trajectory and puncture the needle percutaneously into an optimal position based on their own experience [16]. Training of PETD for inexperienced surgeons is often frustrated by repeated fluoroscopy with increasing punctures [17], which extend the operation time and increase the radiation exposure. Obviously, radiation hazard is a great concern in current clinical practice [18–20]. Furthermore, major complications such as neurovascular injury and incomplete decompression may occur during the learning period [21–23], which will definitely destroy the confidence of the inexperienced surgeons.

Visualized three-dimensional trajectory and guided punctures may help improve the training of PETD. Our previous studies have demonstrated that isocentric navigation was effective in planning definite trajectory and guiding puncture with radiation reduction [24]. As a spine endoscopy-training center, hundreds of inexperienced surgeons were trained in our single center for years. Thus, we aimed to investigate whether isocentric navigation would be beneficial in PETD training program for inexperienced surgeons.

## 2. Materials and Methods

### 2.1. Specimens

The study was approved by the local Institutional Review Board of Shanghai Tenth People's Hospital. All cadaver specimens had no obvious lumbar vertebra deformity, no traumatic defects under fluoroscopy, and no previous lumbar surgery. All operating procedures followed the local cadaver management standards, and the manuscript followed the reporting guideline.

### 2.2. Isocentric Navigation

**Figure 1:**
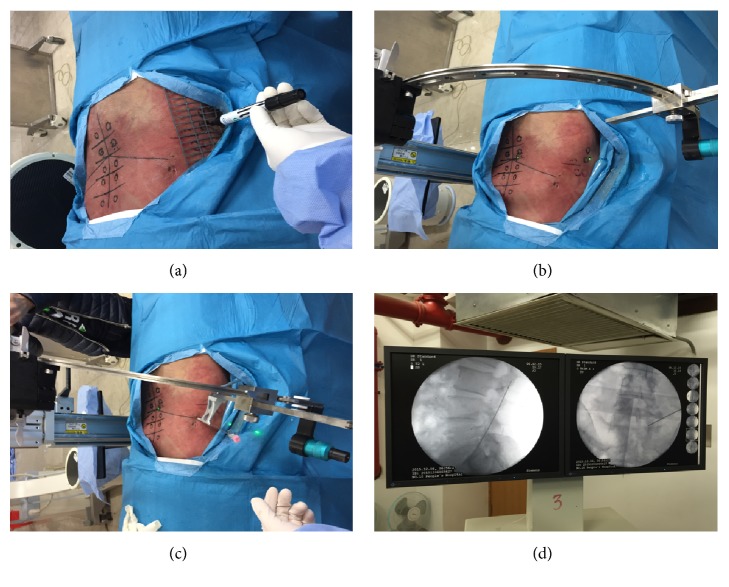
(a) The radiopaque surface locator was attached to the surface of the cadaver to identify the bony landmarks under the intraoperative fluoroscopy. (b) Modifying the arch-guided device to make the vertical beam onto the posterior projection of the puncture target and make the lateral beam onto the lateral projection of the puncture target. (c) Puncture needle was inserted directly to reach the target point along the puncture cannula. (d) Puncture needle was located on the medial pedicle margin in the anteroposterior view and at superior articular process of lower vertebrae on the lateral view.

Isocentric navigation system includes surface locator, puncture needle, and arch-guided device. The radiopaque surface locator is made up of 19 horizontal rods and 4 longitudinal rods [25, 26]. Each horizontal rod is about 9 cm, whereas each longitudinal rod is about 18 cm. There are some different small shape-markers on the horizontal rods whose interval was 1 cm. The location principle of surface locator is to mark the bony landmarks of the body by the surrounding rod and shape-markers. The arch-guided device is mainly composed of a fixed block, a slider, a quarter arch, a guider rod, a needle guider, and 2 beam generators. The theory of arch-guided device is that once the puncture target is located at the center of the virtual sphere forged by the quarter arch, which is achieved by the 2 beam generators, we can reach the puncture target precisely through any radius of the virtual sphere. The surface locator of isocentric navigation is used to accurately and rapidly position the puncture target, and the arch-guided device is used to keep the puncture target at the center of the virtual sphere and assist puncture [24]. The cadavers were placed in prone position on the operation table. The surface locator was used to identify the puncture target and draw some markers on the cadaver to help the surgeon identify the bony landmarks of the cadaver under fluoroscopy (Figure 1(a)). Then the arch-guided device was used to identify the entry point and appropriate trajectory (Figure 1(b)). After that, the puncture needle was installed in the needle guider. Finally, the puncture needle was inserted directly to reach the target point along the puncture cannula (Figure 1(c)).

### 2.3. Grouping and Procedure

As a spine endoscopy-training center, we undertook an academic activity to help the inexperienced surgeons master PETD on cadavers with or without isocentric navigation in Shanghai Tenth People's Hospital from Dec. 2015 to June 2016. There were 117 young surgeons from orthopedic department, neurosurgery department, and department of anesthesia and pain medicine accepting professional training. A total of 117 young surgeons were divided into Group A (n=58) and Group B (n=59) randomly. First, the experienced surgeons interpreted and operated the traditional location and puncture process on the cadaver. Then, inexperienced surgeons in Group A learned the traditional process at the level of L2/L3, L3/L4, L4/L5, and L5/S1 on the right side of the cadaver. Puncture times, fluoroscopy times, exposure time (s), and radiation dose (mSv) were recorded. Next, the experienced surgeons interpreted the isocentric navigation details and demonstrated the location process with the help of surface locator and the puncture process using the arch-guided device on the cadavers. Then, young surgeons in Group B repeated the teaching processes at the level of L2/L3, L3/L4, L4/L5, and L5/S1 on the left side of the cadaver. Puncture times, fluoroscopy times, exposure time(s), and radiation dose (mSv) were also recorded. During the operation, the cadavers were placed on operation table in prone position, and C-arm X-ray machine (ARCADIS, Varic, Siemens) accomplished intraoperative fluoroscopy. The surface locator was used for preoperative location, with which the position of lumbar spinous process, pedicle, intervertebral space, target point, and articular process were confirmed and marked. Intervertebral foramen and intervertebral space were also marked on the body surface laterally (Figure 1(a)). A satisfied puncture was defined as puncture needle located on the medial pedicle margin on anteroposterior fluoroscopy and at superior articular process of lower vertebrae on the lateral fluoroscopy (Figure 1(d)).

### 2.4. Observational Parameters

We recorded and analyzed the puncture times, fluoroscopy times, exposure time (s), and radiation dose (mSv). JB4020X-*γ* personal radiation alarm apparatus (Shanghai Jing Bo Industry & Trade Co., Ltd.) was used to detect the accumulated radiation dose for each segment.

### 2.5. Questionnaire

We conducted a questionnaire to survey the learning of PETD, which was done by 117 young surgeons before our training program. After that, we made another questionnaire to get young surgeons' feedback about the training program.

### 2.6. Statistical Analysis

The software package SPSS 12.0 (SPSS Corporation, USA) was used for statistical analysis. The statistic was demonstrated as mean ± SD. ANOVA test was used to compare the difference of quantitative data, and Chi-square test was used to analyze numerical data. P <0.05 was regarded as statistical significance.

## 3. Results

**Figure 2:**
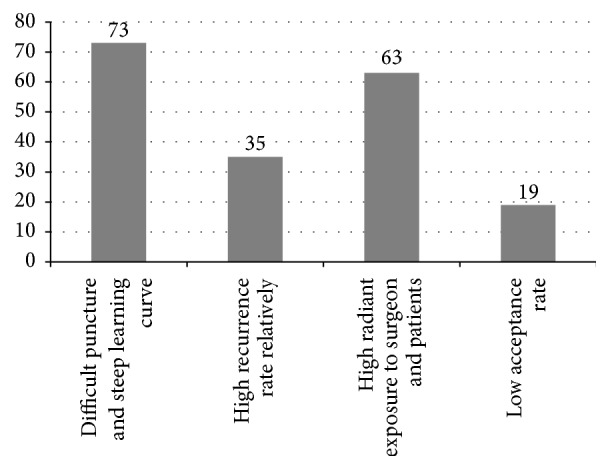
The consequence of the 9th item.

Our designed questionnaire (Table 1) that was conducted before the training program consisted of 15 items. The feedback survey (Table 2) was accomplished in a week after our training program by 117 young surgeons. We focused on the 9th, 11th, 12th, and 15th items of the questionnaire accomplished before the program. The 9th item was multiple-choice item, and the other three were single-choice items. The 9th item revealed that 62.4% (n=73) of young surgeons thought that the difficult puncture made PETD generalization difficult, and 53.8% (n=63) agreed that high radiant exposure makes popularization harder (Figure 2). The feedback survey showed that the satisfaction rate for Group A was 86.2%(n=50), which was 96.6% for Group B (n=57) (p<0.05). A total of 91.5% (n=107) young surgeons were satisfied with the training program in total.

A total of 48.3% (n=28) young surgeons thought that is was not that difficult to master PETD after the program in Group A, which was 67.8% (n=40) in Group B (p<0.05) (Table 3). In Group A, 90.0% (n=52) of young surgeons worried about the radiation hazard before the training program, which increased to 91.4% (n=53) after the program (p>0.05) (Table 4). In Group B, there was also no significant difference in worrying about the radiation hazard before and after the training program, which was 83.1% (n=49) and 88.1% (n=52) (p>0.05) (Table 5). A total of 96.6% (n=56) of surgeons in Group A agreed that we should take measures to avoid radiation hazard, which increased to 98.3% (n=57) after the program (p>0.05). As for Group B, there was also no significant difference in agreement rate before (98.3%, n=58) and after (98.3%, n=58) the program (p>0.05) (Table 5).

Accurate equipment, which could assist location and puncture in PETD for inexperienced surgeons, was required by 89.0% (n=51) in Group A and 76.3%(n=45) in Group B before the program, which turned to 89.7% (n=52) in Group A and 93.2%(n=55) in Group B after the program. There was significant difference in whether accurate equipment was required before and after the training program in Group B (p<0.05) (Table 5).

The experienced surgeons demonstrated the puncture at L2/L3 on the right side of one cadaver and then showed the puncture with the help of isocentric navigation at L2/L3 on the left side of the same cadaver. The 117 young surgeons were divided into two groups randomly. There were 58 young surgeons in Group A and 59 young surgeons in Group B. Group A received the traditional puncture at the level of L2/L3, L3/L4, L4/L5, and L5/S1 on the right side of the 15 cadavers. Group B received the puncture with the help of isocentric navigation at the level of L2/L3, L3/L4, L4/L5, and L5/S1 on the left side of the 15 cadavers.

In Group A, the puncture times of L2/L3, L3/L4, L4/L5, and L5/S1 were 5.500±2.066, 8.000±2.726, 8.333±2.920, and 10.826±2.946 (P<0.001). As for fluoroscopy times, they were 13.357±4.069, 18.467±5.462, 19.533±6.243, and 23.500±6.297 for L2/L3, L3/L4, L4/L5, and L5/S1 (P<0.001). The exposure time (s) was significantly different for L2/L3, L3/L4, L4/L5, and L5/S1, which was 13.000±3.595, 17.267±4.431, 18.333±5.038, and 22.929±6.306, respectively (P<0.001). The radiation dose (mSv) we measured was 0.244±0.067, 0.319±0.085, 0.339±0.097, and 0.423±0.116 for L2/L3, L3/L4, L4/L5, and L5/S1 (P<0.001). The above data indicated that the different anatomic structures of different level among L2/L3, L3/L4, L4/L5, and L5/S1 brought different degree of difficulty (Table 6).

In Group B, the puncture times of L2/L3, L3/L4, L4/L5, and L5/S1 were 1.733±0.799, 1.933±0.961, 1.867±0.640, and 2.286±0.994 (P=0.364). Besides, there was no significant difference in fluoroscopy times (P=0.532), which were 5.867±2.031, 6.000±2.035, 6.400±1.352, and 6.857±2.349 for L2/L3, L3/L4, L4/L5, and L5/S1. As for exposure time (s), it was 5.867±2.031, 6.000±2.035, 6.400±1.352, and 6.786±2.225 for L2/L3, L3/L4, L4/L5, and L5/S1 (P=0.575). The radiation dose (mSv) of L2/L3, L3/L4, L4/L5, and L5/S1 was 0.106±0.035, 0.111±0.035, 0.120±0.026, and 0.126±0.041 (P=0.389). The above data indicated that isocentric navigation could minimize the influence of different levels in PETD surgery (Table 7).

As for intergroup comparison, puncture time for Group A was 8.034±3.117, and it was 1.949±0.860 for Group B (P<0.001). Also, the isocentric navigation could decrease the fluoroscopy times, exposure time (s), and radiation dose (mSv) significantly (P<0.001) (Table 8).

## 4. Discussion

PETD was growing rapidly over the past decades [27]. Surgeons from orthopedic subspecialty [28], neurosurgery subspecialty [29], and anesthesia and pain subspecialty [30] have adopted this technique for the management of lumbar disc herniation cases. However, the learning curve of PETD was very steep [13, 14], mainly because surgeons need to puncture the needle percutaneous into an optimal position based on their experience [16], which is a little bit hard for young surgeons from our survey. Moreover, young surgeons tend to be more and more careful of radiant exposure during PETD. According to our survey, 86.3% (n=101) of surgeons worried about the radiation hazard. International Commission on Radiological Protection (ICRP) had recommended radiation limits per year for professionals specialized in body tissues and organs [20]. Our questionnaire revealed that isocentric navigation could improve young surgeons' satisfaction rate in the training program.

A successful PETD mainly includes optimal placement of working channel and complete decompression of oppressed nerve roots. All these surgical procedures rely on cumulative experience, so it leads to a steep learning curve. It may not be a surprise that the learning curve was associated with the success of PETD [31], but it was also closely correlated with recurrent herniation after successful PETD [32]. The good news is that learning curve of PETD can be overcome with suitable patient selection and effective training [33]. However, the training of PETD still remains as a challenge, because inexperienced surgeons have no spatial sense of a three-dimensional trajectory for the initial puncture. This is an inconvenient issue when we conventionally adopted X-ray C-arm machine to assist PETD, because we cannot easily interpret the angles of the planned trajectory on two-dimensional fluoroscopy into three-dimensional angles. Researchers tried to develop new preoperative trajectory planning for PETD on oblique MRI [34], but preoperative MRI is not always consistent with the prone position of intraoperative patient. Others tried to introduce three-dimensional angles of planned trajectory into PETD, but they all failed to transfer these quantified angles into intraoperative punctures [35, 36]. Obviously, isocentric navigation is capable of quantifying three-dimensional angles and helps inexperienced surgeons build spatial sense of their trajectory angle in PETD training, which is consistent with the result that more surgeons in Group B were satisfied with our training.

Among our survey, 97.4% of inexperienced surgeons agreed that there is a need to take measures to avoid radiant hazard during PETD. There are many measures to minimize radiant hazard such as decreasing fluoroscopy time, keeping away from the machine, using low-dose model, and shielding protection [19]. Some researchers [37] have developed a foraminotomy tool to reduce radiation exposure, and their results in experiment group (40.71 ± 6.23 seconds) were promising compared with conventional group (49.20 ± 7.84 seconds). However, only 17.25% reduction of radiation exposure time was achieved in their study. Others [38] tried to apply ultrasound to assist PETD, which only needs 2.9 ± 0.7 seconds of radiation exposure time. It seems to be a very promising technology to assist PETD, but they did not confirm whether ultrasound would introduce another learning curve since most surgeons were already familiar with X-ray C-arm fluoroscopy. Therefore, isocentric navigation is still a very promising technology in PETD training, because it is perfectly compatible with X-ray C-arm fluoroscopy.

In our study, isocentric navigation could decrease puncture times, fluoroscopy times, exposure time, and radiation dose significantly, which is beneficial to the training of PETD for inexperienced surgeons. More importantly, isocentric navigation seems to minimize the difference among different surgical levels. This was an interesting finding, because learning curve of PETD at different surgical level might be quite different [39]. Generally speaking, PETD at L5/S1 level was usually more difficult than that at the other surgical levels, because it might be confronted with high iliac crest, narrow foramen, and enlarged facet joint. Luckily, isocentric navigation is also capable of overcoming these difficulties at L5/S1 level [24]. This will certainly contribute to the confidence of inexperienced surgeons when they get PETD training and overcome the learning curve. In other words, isocentric navigation seems to improve PETD standardization and homogenization for inexperienced surgeons.

There were some limitations that should be noted in this study. First, the self-made questionnaire might not cover all pertinent questions concerning PETD training and we could not find professional scale to directly quantify the psychological burden of radiation concern. Secondly, the trajectory planning of isocentric navigation in this training is a bit primitive. We are still working on how to combine virtual reality with isocentric navigation for PETD, including preoperative planning and intraoperative training.

## 5. Conclusions

Many young surgeons think PETD technology is very hard for them and agree that there should be a novel device to assist PETD. They also considered that there should be more training programs for them to master PETD. The pilot study validates the feasibility and efficacy of isocentric navigation for PETD training, because it can minimize the learning difficulty for all levels with decreased puncture times, fluoroscopy times, exposure times, and radiation dose. Therefore, the isocentric navigation has the potential to improve PETD standardization and homogenization for inexperienced surgeons, which is beneficial for PETD's popularization and generalization.

## Figures and Tables

**Table 1:** Questionnaire.

The radiant exposure of percutaneous endoscopic transforaminal discectomy (PETD) and its influence on learning of PETD	
(1) What is your age?	—
How many years do you work on medicine?	—
(2) What is your academic degree?	
□ Doctor	□ Master
□ Bachelor	□ Associate degree
(3) What is the category of your hospital?	
□ First level of public hospital	□ Second level of public hospital
□ Third level of public hospital	□ Fourth level of public hospital
□ Private hospital	
(4) Which department do you work on?	
□ Orthopedics	□ Department of pain
□ Neurosurgery	□ Department of intervention
□ Department of anesthesia	
(5) What is your professional title?	
□ Director	□ Deputy director
□ Attending	□ Resident
(6) For how many years had you worked on minimally invasive spinal surgery (MISS) before studying PETD?	—
(7) Are you familiar with PETD?	
□ Very familiar	□ Familiar
□ Less familiar	
(8) What is your reason of learning PETD?	
□ Clinical efficacy is not worse than open surgery	□ Highly minimal invasive and shorter time of recovery
□ Propaganda of associated company	□ Required by patients
(9) What is the difficulty of generalization of PETD do you consider?	
□ Difficult puncture and steep learning curve	□ High recurrence rate relatively
□ High radiant exposure to surgeon and patients	□ Low acceptance rate
(10) Do you clearly know about the radiation hazard during the surgery?	
□ Don't know	□ Less clear
□ Clear	□ Very clear
(11) Do you worry about the radiation hazard?	
□ Very worry about it	□ Worry about it
□ Little worry about it	
(12) Do you think there is a need to take measures to avoid radiation hazard during PETD?	
□ No	□ Yes
(13) What measures will you take to avoid radiation hazard during PETD?	
□ Lead barrier	□ Lead suit
□ Lead collar	□ Lead glasses
□ Lead cap	□ Wear a thermoluminescent tablet which could detect radiation dose
(14) If there is a possibility that one day you may abandon PETD during your learning about it, what do you think the reason would be?	
□ Depression caused by repeat puncture	□ At an old age
□ Complaint of long surgical time from patients	□ Too much radiation exposure caused by repeat fluoroscopy
□ Worse clinical efficacy and sever postoperative complication	
(15) Do you think that there is a need for accurate equipment that could assist location and puncture during PETD for young surgeons?	
□ Yes	□ No

**Table 2:** Feedback survey.

Feedback survey after the training program	
(1) Are you satisfied with this training program?	
□ Yes	□ No
(2) Do you agree on that PETD is not that difficult to master?	
□ Yes	□ No
(3) Do you still worry about the radiation hazard?	
□ Very worry about it	□ Worry about it
□ Less worry about it	
(4) Do you think whether there is a need to take measures to avoid radiation hazard during PETD?	
□ No	□ Yes
(5) Do you think if there is a need for accurate equipment that could assist location and puncture during PETD for young surgeons?	
□ Yes	□ No

**Table 3:** Satisfaction rate for the training program and agreement rate on the thought that it is not that difficult to master PETD between Group A and Group B.

Analytic terms	Group A	Group B	P Value
Satisfaction rate	86.2% (n=50)	96.6%(n=57)	<0.05
Agree rate	48.3%(n=28)	67.8%(n=40)	<0.05

**Table 4:** Analysis in Group A.

Analytic terms	Before the program	After the program	P Value
Worry rate	90.0% (n=52)	91.4%(n=53)	0.75
Urge rate	96.6%(n=56)	98.3%(n=57)	0.56
Requirement rate	89.0%(n=51)	89.7%(n=52)	0.77

**Table 5:** Analysis in Group B.

Analytic terms	Before the program	After the program	P Value
Worry rate	83.1% (n=49)	88.1%(n=52)	0.43
Urge rate	98.3%(n=58)	98.3%(n=58)	1.00

Requirement rate	76.3%(n=45)	93.2%(n=55)	<0.05

**Table 6:** The data we recorded and analyzed in Group A.

Outcomes	L2/3	L3/4	L4/5	L5/S1	P value
Puncture times	5.500±2.066	8.000±2.726	8.333±2.920	10.286±2.946	<0.001
Fluoroscopy times	13.357±4.069	18.467±5.462	19.533±6.243	23.500±6.297	<0.001
Exposure time (s)	13.000±3.595	17.267±4.431	18.333±5.038	22.929±6.306	<0.001
Radiation dose (mSv)	0.244±0.067	0.319±0.085	0.339±0.097	0.423±0.116	<0.001

**Table 7:** The data we recorded and analyzed in Group B.

Outcomes	L2/3	L3/4	L4/5	L5/S1	P value
Puncture times	1.733±0.799	1.933±0.961	1.867±0.640	2.286±0.994	0.364
Fluoroscopy times	5.867±2.031	6.000±2.035	6.400±1.352	6.857±2.349	0.532
Exposure time (s)	5.867±2.031	6.000±2.035	6.400±1.352	6.786±2.225	0.575
Radiation dose (mSv)	0.106±0.035	0.111±0.035	0.120±0.026	0.126±0.041	0.389

**Table 8:** The average values of recorded data were analyzed between Group A and Group B.

Outcomes	Group A	Group B	P value
Puncture times	8.034±3.117	1.949±0.860	<0.001
Fluoroscopy times	18.724±6.526	6.271±1.955	<0.001
Exposure time (s)	17.879±5.944	6.254±1.917	<0.001
Radiation dose (mSv)	0.331±0.110	0.116±0.035	<0.001

## Data Availability

The data is available via the following link: https://www.dropbox.com/sh/2qb8xu0hxadogc2/AAArOrPq3OFjfRmhWxKYu0zea?dl=0 or from the corresponding author upon request.
